# Dose Proportionality of Fentanyl Buccal Tablet in Healthy Japanese Volunteers

**DOI:** 10.1111/j.1753-5174.2008.00007.x

**Published:** 2008-09

**Authors:** Mona Darwish, Kenneth Tempero, John G Jiang, Jeffrey Thompson, Philip G Simonson

**Affiliations:** * Cephalon, Inc.Frazer, PA, USA; † 1901 Lake Road, Wayzata, MN, USA

**Keywords:** Fentanyl Buccal Tablet (FBT), Dose Proportionality, Pharmacokinetics, Japanese

## Abstract

**Objective:**

This study was conducted to assess the dose proportionality, safety, and tolerability of fentanyl buccal tablet (FBT) in Japanese volunteers.

**Methods:**

Healthy, opioid-naive Japanese adults received single-dose FBT 100, 200, 400, and 800 µg in a randomized, open-label, crossover fashion. Naltrexone was given to minimize the opioid effects of fentanyl. Peak serum fentanyl concentration (C_max_), time to C_max_ (t_max_), area under the serum fentanyl concentration-time curve (AUC) from time 0 to infinity (AUC_0–∞_), and AUC from 0 to the last quantifiable concentration (AUC_0–last_) were summarized using descriptive statistics. Dose proportionality was claimed if the ln-ln plots of C_max_, AUC_0–∞_, and AUC_0–last_ vs. dose were linear and the 90% confidence intervals (CI) of the slopes were within 0.8927 and 1.1073. The safety population comprised volunteers who received ≥1 FBT.

**Results:**

Twenty-five volunteers were enrolled, 23 were included in the safety population (mean age 35.3 years), and 19 completed the study. The assessment of dose proportionality did not meet the statistical criteria (slope [90% CI]: 0.9118 [0.8601, 0.9635] for C_max_, 1.0756 [1.0377, 1.1136] for AUC_0–∞_, and 1.0992 [1.0677, 1.1307] for AUC_0–last_). However, the increase in systemic exposure with dose appeared linear, and a post hoc analysis of partial AUCs from time 0 to 8, 12, 18, and 24 hours supported dose proportionality. Median t_max_ of 90 minutes (range 30–180 minutes) was independent of dose. Adverse events (AEs) were mild or moderate. The most frequent AEs were nausea (N = 9), dizziness (N = 8), headache (N = 6), somnolence (N = 6), dyspepsia (N = 5), and vomiting (N = 3). No application-site or serious AEs were reported.

**Conclusions:**

Systemic exposure to FBT was approximately dose proportional across the range 100 µg to 800 µg in healthy Japanese adults. Adverse events were mild or moderate.

## Introduction

Fentanyl buccal tablet (FBT; *FENTORA*®, Cephalon, Inc., Frazer, PA, USA) is approved for marketing in the United States for the management of breakthrough pain in patients with cancer who are already receiving and who are tolerant to around-the-clock opioid therapy for their underlying persistent cancer pain [1]. FBT employs OraVescent® technology to enhance the rate and extent of fentanyl absorption across the buccal mucosa [2].

Assessment of dose proportionality can help predict patients’ response to changes in dose. Drugs that exhibit linear pharmacokinetics usually provide a predictable systemic exposure when a dose is increased or decreased. The objective of this study was to investigate the pharmacokinetics, including dose proportionality, and the safety and tolerability of FBT 100 µg to 800 µg in healthy Japanese volunteers. The study was conducted to support the submission of a new drug application for FBT in Japan.

## Methods

### Study Population

Healthy Japanese men and women living in the United States, aged 20 to 55 years with a body mass index of 17.6 to 29 kg/m^2^, were eligible for the study if they had clinically normal findings based on medical history, physical examination, and laboratory tests (serum chemistry, hematology, urinalysis, human immunodeficiency virus test screen, hepatitis B surface antigen screen, and hepatitis C antibody screen). Women of childbearing potential were required to have a negative serum pregnancy test result and to practice a reliable form of contraception or abstinence for 2 weeks before screening and throughout the study. Eligible volunteers were required to have a negative urine drug test result (cocaine, opiates, cannabinoids, alcohol) at the time of screening and at check-in for each study phase. Volunteers were excluded if they had smoked more than 10 cigarettes per day within 3 months of the first scheduled dose of FBT. Volunteers were prohibited from using over-the-counter medications (including herbal supplements) and prescription medications within 7 days and 14 days, respectively, of the first dose of FBT and throughout the study. Drugs or substances known to strongly induce cytochrome P450 (CYP) enzymes were prohibited within 30 days of the start of the study; drugs or substances known to strongly inhibit CYP enzymes were prohibited within 10 days of the start of the study.

### Study Design

This study was conducted at a single center in Hawaii (Radiant Research, Honolulu). The protocol was approved by the Institutional Review Board of Quorum Review, Inc. All volunteers provided written informed consent.

In this open-label, randomized, crossover study, volunteers received a single dose of FBT 100 µg, 200 µg, 400 µg, and 800 µg in 4 treatment phases separated by at least 7 days of washout. These doses were selected because previous pharmacokinetics studies indicated that FBT 100 µg to 800 µg would result in serum fentanyl concentrations sufficient to achieve study objectives with minimal risk to healthy volunteers administered naltrexone [3]. Because healthy volunteers are not opioid tolerant, oral naltrexone hydrochloride 50 mg (Barr Pharmaceuticals, Inc., Pomona, NY) was given 15 hours and 3 hours before FBT administration in all study phases to minimize the opioid receptor–mediated effects of fentanyl. Naltrexone hydrochloride was also given 12 hours after FBT administration for the treatment phases with FBT 400 µg and 800 µg. Coadministration of naltrexone with fentanyl would not be expected to affect the pharmacokinetics of fentanyl because fentanyl is a substrate of CYP3A4 [4], and naltrexone is not an inhibitor or inducer of CYP3A4 [5].

Fasting volunteers self-administered FBT by placing the tablet buccally above a molar tooth and allowing it to dissolve for 10 minutes. If any portion of the tablet remained at that time, volunteers were instructed to gently massage the adjacent cheek area for 5 minutes to facilitate dissolution. (It is important to note that massage is no longer recommended in the FBT prescribing information [1].) Any tablet material remaining after the 5-minute massage (as verified by study center personnel) was allowed to disintegrate on its own.

### Dwell Time Assessment

Dwell time, defined as the time between tablet placement and complete disappearance of tablet residue, was visually verified and recorded by study personnel.

### Sample Collection

Venous blood samples (5 mL) were collected before and 0.25, 0.5, 0.75, 1, 1.5, 2, 3, 4, 6, 8, 10, 12, 18, 24, 30, and 36 hours after placement of FBT for serum fentanyl assay. Samples were allowed to clot at room temperature. They were then centrifuged at 2,500 revolutions per minute for 15 minutes at 4°C to separate the serum, which was transferred to clear polypropylene containers, and stored at −20°C until assayed.

### Analytical Methods

Serum samples were analyzed for fentanyl concentrations using a validated assay for high-performance liquid chromatography with tandem mass spectrometric detection (HPLC-MS/MS; PE Sciex API 3,000 mass spectrometer). The analyte and internal standard (d_5_-fentanyl) were extracted from serum by liquid-liquid extraction under basic conditions. Serum extracts were evaporated to dryness and reconstituted for injection onto the HPLC-MS/MS. Detection was accomplished using multiple-reaction monitoring in positive ion mode. The linear range for the assay was 10 to 5,000 pg/mL (0.01–5.0 ng/mL), and the lower limit of quantitation was 50 pg/mL (0.05 ng/mL). The interassay precision (% coefficient of variation) of quality control samples was ≤3.6%, and interassay accuracy ranged from 96.3% to 100.7%.

### Pharmacokinetic Analysis

The maximum serum fentanyl concentration (C_max_) was obtained by direct inspection of the data without interpolation; t_max_ was defined as the time to reach C_max_. The area under the serum fentanyl concentration-time curve (AUC) from time zero to the time of the last quantifiable concentration (C_last_; AUC_0–last_) was determined using linear trapezoidal summation. AUC from time zero extrapolated to infinity (AUC_0–∞_) was calculated as AUC_0–last_ + C_last_/λ_z_, where λ_z_ was the terminal phase elimination rate constant (from the negative slope of the linear regression of log concentration over time in a terminal phase that included at least 3 time points). AUC_0–∞_ values were dropped from the analysis if the extrapolated area was >20% of the total AUC_0–∞_. Half-life (t_½_) was calculated as ln(2)/λ_z_. Serum fentanyl concentrations below the limit of detection were set to zero if they occurred at the beginning of the profile and were considered missing if they occurred at the last measurable concentration. Pharmacokinetic parameters for fentanyl were estimated using non-compartmental methods with WinNonlin® Professional software Version 4.0 or higher (Pharsight Corporation, Mountain View, CA).

Because the analysis could be biased by the fentanyl concentrations that fell below the limit of detection at the low doses, post hoc analyses were performed to assess dose proportionality using partial AUCs, which were calculated at 8 hours (AUC_0–8_), 12 hours (AUC_0–12_), 18 hours (AUC_0–18_), and 24 hours (AUC_0–24_). Calculation of these AUCs minimized the percentage of the extrapolated area contributing to values for AUC_0–∞_.

### Safety and Tolerability Assessments

The safety and tolerability of FBT was assessed by monitoring adverse events (AEs), clinical laboratory test results, vital signs, ECGs, and physical examination findings, including evaluation of the oral mucosa. Clinical laboratory tests (serum chemistry, hematology, and urinalysis), physical examination including vital signs, and a 12-lead ECG were performed at screening and the end of the study. Vital signs (respiratory rate, heart rate, and blood pressure) were evaluated before and through 36 hours after each dose of study medication was administered. Pulse oximetry was monitored continuously for 4 hours after FBT placement, as well as any time the volunteers attempted to sleep during the 12 hours after tablet placement. In each treatment phase, the oral mucosa was examined by the investigator 4 hours after placement of FBT.

### Statistical Analyses

The sample size of volunteers was not based on a statistical power calculation but reflects the typical number of volunteers enrolled in pharmacokinetic studies. Descriptive statistics were used to summarize the pharmacokinetic parameters for FBT by dose. The slopes were obtained for natural log (ln)-transformed pharmacokinetic parameters vs. ln(dose). The primary methodology used to assess dose proportionality from 100 µg to 800 µg was a comparison of the 90% confidence interval (CI) of the slopes with a criterion CI, based on a power model [6]. A mixed-effect model was used on ln-transformed AUC_0–last_, AUC_0–∞_, and C_max_. The model included ln-transformed dose, treatment order, and period as fixed effects, and intercept and volunteer as random effects. The criterion interval for testing the slope of the power model β = 1 was as follows: [1 + (ln(0.80)/ln(high/low)], 1 + [ln(1.25)/ln(high/low)], where high = 800 μg (highest dose) and low = 100 μg (lowest dose). It resulted in (0.8927, 1.1073). Dose proportionality was concluded if the plot of pharmacokinetic parameter vs. dose indicated linearity (i.e., slope = 1) and the 90% CI for the slope fell within the range 0.8927 to 1.1073.

Analyses of variance were performed for pairwise comparisons on the dose-normalized and ln-transformed AUC_0–last_, AUC_0–∞_, and C_max_. Treatment order, treatment, and period were fixed effects, and volunteer nested within treatment order was a random effect. The statistical analyses were performed with SAS PROC MIXED software.

In the post hoc analysis, the same statistical analysis model for dose proportionality used on AUC_0–∞_ and AUC_0–last_ was applied to the partial AUC values for AUC_0–8_, AUC_0–12_, AUC_0–18_, and AUC_0–24_.

The pharmacokinetic population comprised volunteers who completed ≥2 periods of the study. The safety analysis population in this study comprised all volunteers who received ≥1 dose of FBT.

## Results

### Study Population

Twenty-five volunteers were enrolled in the study; 23 (2 men, 21 women) aged 20 to 51 years (Table 1) were included in both the pharmacokinetic and safety populations, and 19 completed the study. Six volunteers were discontinued from the study: 4 because of withdrawal of consent (1 from each treatment order) and 2 because of adverse events (AEs) that occurred after naltrexone administration but before FBT administration. The pharmacokinetic and safety analyses excluded the 2 volunteers who did not receive FBT.

**Table 1:** Demographic variables

	N = 23
Age, year (mean [SD])	35 (8)
Sex, female (N [%])	21 (91)
Weight, kg (mean [SD])	57.4 (10.7)
Height, cm (mean [SD])	162.3 (8.4)
Body mass index, kg/m^2^ (mean [SD])	21.8 (3.6)

SD = standard deviation.

### Pharmacokinetic Findings

**Figure 1:**
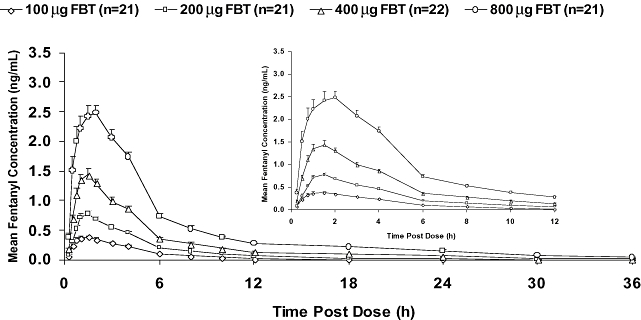
Mean (standard error of the mean) serum fentanyl concentrations after administration of FBT 100, 200, 400, and 800 µg.

The mean serum fentanyl concentration-time curves for FBT 100, 200, 400, and 800 µg are illustrated in Figure 1. After the 100 and 200 µg doses, serum fentanyl decreased from peak concentrations in a biphasic manner, but the later portions of these profiles tended to fall below quantifiable limits in some volunteers within 8 hours after FBT administration and before the terminal elimination phase was identifiable. Following the 400 and 800 µg doses, fentanyl concentrations decreased from peak values in a triphasic manner.

**Figure 2:**
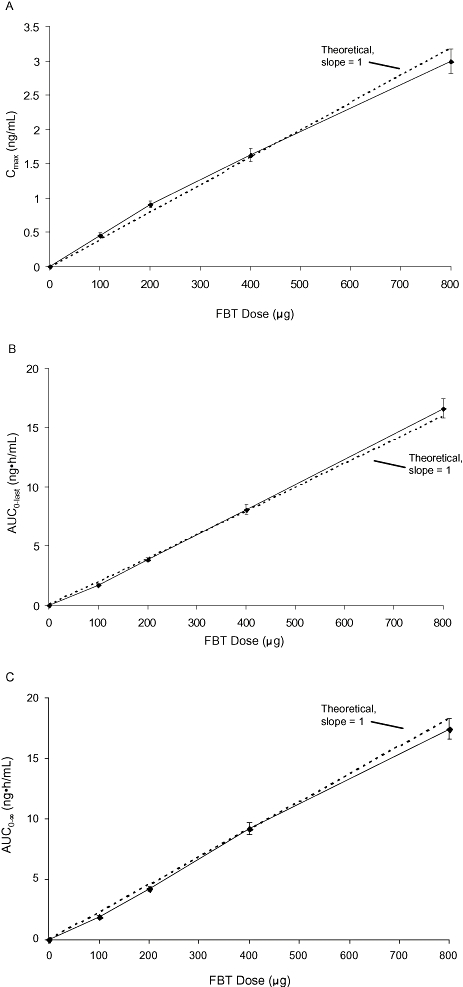
Relationship between mean (standard error of the mean) C_max_ (A), AUC_0–last_ (B), and AUC_0–∞_ (C) vs. Dose of FBT. For dose proportionality, the prespecified bounds for the 90% CIs of the slopes were 0.8927 to 1.1073. The slopes (90% CI) were 0.9118 (0.8601, 0.9635) for C_max_, 1.0756 (1.0377, 1.1136) for AUC_0–∞_, and 1.0992 (1.0677, 1.1307) for AUC_0–last_. AUC_0–last_ = area under the serum fentanyl concentration-time curve (AUC) from time zero to the last quantifiable concentration; AUC_0–∞_ = AUC_0_ to infinity; C_max_ = maximum observed serum fentanyl concentration.

Pharmacokinetic parameters for the fentanyl doses are listed in Table 2. T_max_ occurred at a median of 90 minutes (range 30–180 minutes) for all doses. Mean C_max_, AUC_0–∞_, and AUC_0–last_ increased with increasing doses of FBT (Table 2, Figure 2). Mean t_½_ of FBT increased with dose up to 400 µg; however, for the 100 and 200 µg doses, as previously stated, fentanyl concentrations fell below quantifiable limits early, which prohibited accurate estimation of the terminal t_½_. Mean t_½_ did not increase for the 400 to 800 µg doses of FBT.

**Table 2:** Pharmacokinetic parameters after a single dose of FBT 100, 200, 400, and 800 µg

	FBT dose				
		100 µg	200 µg	400 µg	800 µg
Parameter	Statistic	(N = 21)	(N = 21)	(N = 22)	(N = 21)
C_max_ (ng/mL)	Mean	0.45	0.91	1.62	2.99
	SD	0.17	0.22	0.43	0.80
AUC_0–∞_ (ng·h/mL)*	Mean	1.86	4.21	9.18	17.44
	SD	0.47	0.95	2.24	3.88
AUC_0–last_ (ng·h/mL)	Mean	1.71	3.84	8.07	16.60
	SD	0.45	0.81	1.93	3.80
t_max_ (min)	Median	90	90	90	90
	Range	30, 180	30, 180	30, 120	30, 180
t_½_ (h)*	Mean	2.60	5.56	10.44	10.06
	SD	0.940	3.236	3.576	2.954

* N = 16, 19, 18, and 20 for 100, 200, 400, and 800 µg, respectively.

SD = standard deviation; C_max_ = maximum observed serum fentanyl concentration; AUC_0–∞_ = area under the serum fentanyl concentration-time curve from time zero to infinity; AUC_0–last_ = AUC_0_ to the time of the last quantifiable concentration; t_max_ = time to C_max_; t_½_ = elimination half-life.

### FBT Dose Proportionality

Statistical analysis showed that the slopes of the plots for ln-transformed C_max_, AUC_0–∞_, and AUC_0–last_ vs. ln-transformed dose were all close to 1, but the 90% CIs of the slopes were not completely contained within the predefined range for dose proportionality (0.8927, 1.1073). The slopes (90% CIs) were 0.9118 (0.8601, 0.9635) for C_max_, 1.0756 (1.0377, 1.1136) for AUC_0–∞_, and 1.0992 (1.0677, 1.1307) for AUC_0–last_. Dose proportionality was not observed by analysis of variance performed for pairwise comparisons on ln-transformed and dose-normalized AUC_0–last_, AUC_0–∞_, and C_max_.

The statistical analysis may have failed to show dose proportionality because, since serum fentanyl levels fell below the limit of quantitation after 18 hours after the administration of FBT 100 µg and 200 µg in some volunteers, AUC_0–∞_, AUC_0–last_, and t_½_ values were probably underestimated. For this reason, a post hoc analysis was performed on partial AUCs.

In the post hoc analysis of partial AUCs, the criteria for dose proportionality were met. The slopes of ln-transformed AUC vs. ln-transformed dose were close to 1 and their 90% CIs fell within the predefined range required for dose proportionality: AUC_0–8_ (slope = 0.95 [90% CI = 0.9188, 0.9737]), AUC_0–12_ (0.96 [0.9323, 0.9873]), AUC_0–18_ (0.99 [0.9567, 1.0135]), and AUC_0–24_ (1.01 [0.9823, 1.0423]). An analysis of variance was also performed for pairwise comparisons on ln-transformed and dose-normalized AUC_0–8_, AUC_0–12_, AUC_0–18_, and AUC_0–24_. Dose proportionality was found for AUC_0–18_ and AUC_0–24_, but not for AUC_0–8_ or AUC_0–12_ over the 100 to 800 µg dose range.

### Buccal Dwell Time

The mean buccal dwell times for FBT were 48.95, 59.24, 59.27, and 68.14 minutes for the 100, 200, 400, and 800 µg doses, respectively.

### Safety and Tolerability

As stated previously, naltrexone was administered to these healthy volunteers to minimize the opioid effects of fentanyl. In the safety analysis set, 18 volunteers had ≥1 AE, and all AEs were mild or moderate. No volunteers in the safety analysis set had serious AEs or discontinuations resulting from AEs. The most frequent AEs are summarized in Table 3. One report of gingival pain (200 µg dose) was the only AE associated with the oral mucosa, and it was considered by the investigator to be related to the study regimen. There was no evidence of a relationship between AEs and FBT dose, except for dizziness, which was reported more frequently at the 800 µg dose. A single report of vasovagal syncope was not considered by the investigator to be related to the study drug. There were no clinically significant changes in laboratory measures, ECG parameters, or physical examination findings from screening to the end of the study, nor were there any significant changes in vital signs at 2 hours (time point close to the median t_max_ of 90 minutes) or 4 hours after administration of any of the doses.

**Table 3:** Adverse events occurring in ≥5% of volunteers at any dose of FBT 100 µg to 800 µg*

	FBT dose				
	100 µg	200 µg	400 µg	800 µg	Total
	(N = 21)	(N = 21)	(N = 22)	(N = 21)	(N = 23)
Adverse event	N (%)	N (%)	N (%)	N (%)	N (%)
Dizziness	2 (9.5)	2 (9.5)	2 (9.1)	4 (19.0)	8 (34.8)
Dyspepsia	2 (9.5)	3 (14.3)	1 (4.5)	2 (9.5)	5 (21.7)
Headache	2 (9.5)	1 (4.8)	3 (13.6)	2 (9.5)	6 (26.1)
Nausea	3 (14.3)	4 (19.0)	2 (9.1)	2 (9.5)	9 (39.1)
Somnolence	1 (4.8)	2 (9.5)	2 (9.1)	2 (9.5)	6 (26.1)
Vomiting	0 (0)	3 (14.3)	1 (4.5)	0 (0)	3 (13.0)

* Volunteers who had multiple episodes of a given adverse event during a treatment phase are counted once.

## Discussion

The primary objective of this study was to evaluate the dose proportionality of fentanyl C_max_, AUC_0–last_, and AUC_0–∞_ across the dose range of FBT 100 to 800 µg in healthy Japanese volunteers. These parameters appeared to be approximately dose proportional, though they did not meet the statistical criteria for dose proportionality. Post hoc analysis of partial areas from time zero to serum sampling times at 8, 12, 18, and 24 hours (AUC_0–8_, AUC_0–12_, AUC_0–18_, and AUC_0–24_) showed that all of these truncated areas were proportional to dose. The mean buccal dwell times for FBT ranged from 49 to 68 minutes; however, no association has been found between variations in dwell time and FBT pharmacokinetic parameters and early exposure as measured by C_max_, t_max_, and AUC_0–tmax′_[7].

Safety and tolerability findings would not be expected to reflect those in patients because the healthy volunteers in this study were administered oral naltrexone to minimize the opioid effects of fentanyl. All AEs were mild or moderate. The most common AEs were nausea, dizziness, headache, somnolence, dyspepsia, and vomiting, a finding similar to that previously reported in healthy volunteers administered FBT and naltrexone [3,8–10].

A number of elements intrinsic to open-label studies in healthy individuals limit the interpretation of results, such as differences in drug metabolism in healthy persons compared with patients who have medical conditions, such as hepatic or renal impairment. Unlike healthy individuals, patients with chronic medical conditions may be taking multiple pharmacologic therapies that could alter drug metabolism.

In conclusion, although the primary results from this study did not meet the statistical criteria for dose proportionality over the FBT dose range of 100 to 800 µg, a post hoc analysis examining AUCs for 0–8, 0–12, 0–18, and 0–24 hours supported dose proportionality.
